# BILE DUCT INJURY REPAIR IN A PATIENT WITH *SITUS INVERSUS TOTALIS*

**DOI:** 10.1590/0102-672020240002e1795

**Published:** 2024-03-18

**Authors:** José Donizeti MEIRA-JÚNIOR, Javier RAMOS-ARANDA, Javier CARRILLO-VIDALES, Erik Rodrigo VELÁSQUEZ-CORIA, Miguel Angel MERCADO, Ismael DOMINGUEZ-ROSADO

**Affiliations:** 1 Universidade de São Paulo, Digestive Surgery Division, Department of Gastroenterology - São Paulo (SP), Brazil;; 2 Instituto Nacional de Ciencias Médicas y Nutrición Salvador Zubirán, Hepatopancreatobiliary Surgery Division, Mexico City, Mexico.

**Keywords:** Cholecystectomy, Bile Duct Diseases, Situs Inversus, Cholecystitis, Cholangitis, Postoperative Complications, Colecistectomia, Doenças dos Ductos Biliares, Situs Inversus, Colecistite, Colangite, Complicações Pós-Operatórias

## Abstract

**BACKGROUND:**

Bile duct injury (BDI) causes significant sequelae for the patient in terms of morbidity, mortality, and long-term quality of life, and should be managed in centers with expertise. Anatomical variants may contribute to a higher risk of BDI during cholecystectomy.

**AIMS:**

To report a case of bile duct injury in a patient with situs inversus totalis.

**METHODS:**

A 42-year-old female patient with a previous history of situs inversus totalis and a BDI was initially operated on simultaneously to the lesion ten years ago by a non-specialized surgeon. She was referred to a specialized center due to recurrent episodes of cholangitis and a cholestatic laboratory pattern. Cholangioresonance revealed a severe anastomotic stricture. Due to her young age and recurrent cholangitis, she was submitted to a redo hepaticojejunostomy with the Hepp-Couinaud technique. To the best of our knowledge, this is the first report of BDI repair in a patient with situs inversus totalis.

**RESULTS:**

The previous hepaticojejunostomy was undone and remade with the Hepp-Couinaud technique high in the hilar plate with a wide opening in the hepatic confluence of the bile ducts towards the left hepatic duct. The previous Roux limb was maintained. Postoperative recovery was uneventful, the drain was removed on the seventh post-operative day, and the patient is now asymptomatic, with normal bilirubin and canalicular enzymes, and no further episodes of cholestasis or cholangitis.

**CONCLUSIONS:**

Anatomical variants may increase the difficulty of both cholecystectomy and BDI repair. BDI repair should be performed in a specialized center by formal hepato-pancreato-biliary surgeons to assure a safe perioperative management and a good long-term outcome.

## RESUMO

**RACIONAL:**

As lesões de via biliar (LVB) impõem sequelas significativas ao paciente em termos de morbidade, mortalidade e qualidade de vida a longo prazo, devendo ser manejadas em centros especializados. Variantes anatômicas podem contribuir para um maior risco de LVB durante colecistectomia.

**OBJETIVOS:**

Relatar paciente com lesão de via biliar associado a *situs inversus totalis.*

**MÉTODOS:**

Paciente do sexo feminino, 42 anos, com histórico prévio de *situs inversus totalis* e LVB inicialmente reparada simultaneamente à lesão, há 10 anos, por um cirurgião não especializado. Ela foi encaminhada a um centro especializado devido a episódios recorrentes de colangite e um padrão laboratorial colestático. Colangiressonância revelou uma grave estenose anastomótica. Devido à sua idade jovem e colangites recorrentes, foi submetida a uma revisão cirúrgica da hepaticojejunostomia com técnica de Hepp-Couinaud. Até onde sabemos, este é o primeiro relato de reparo de LVB em um paciente com *situs inversus totalis*.

**RESULTADOS:**

A hepaticojejunostomia realizado prèviamente foi desfeita e refeita empregando a técnica de Hepp-Couinaud, alta na placa hilar, com uma ampla abertura na confluência dos ductos biliares em direção ao ducto hepático esquerdo. A alça de roux anterior foi mantida. A recuperação pós-operatória transcorreu sem intercorrências, o dreno foi removido no sétimo dia pós-operatório, e a paciente está agora assintomática, com bilirrubina e enzimas canalículares normais, e sem mais episódios de colestase ou colangite.

**CONCLUSÕES:**

Variantes anatômicas podem aumentar a dificuldade tanto da colecistectomia quanto do reparo de LVB, o qual deve ser realizado em um centro especializado por cirurgiões hepatobiliares para garantir um manejo perioperatório seguro e um bom resultado a longo prazo.

## INTRODUCTION

Bile duct injury (BDI) is the most serious complication of laparoscopic cholecystectomy. Despite a relatively low incidence (0.3-1.5%)^21^ ^,^ ^26^, it is a major healthcare burden, considering the high frequency of cholecystectomies performed worldwide. Concomitant vascular injuries are present in up to 32% of patients with a post-cholecystectomy biliary injury^24^, which makes its management even more difficult. BDIs are a surgical challenge, impose significant sequelae for the patient in terms of morbidity, mortality, and long-term quality of life^3^ ^,^ ^8^ ^,^ ^16^, and should be managed in centers with expertise^23^. Risk factors that could contribute to a higher risk of BDI during cholecystectomy include acute cholecystitis, severe obesity, previous surgery on the biliary tract, underlying liver disease, and the several anatomical variants of the biliary tract^4^ ^,^ ^20^.

Situs inversus totalis (SIT) is a rare autosomal recessive congenital disorder, affecting between 1:10.000 to 1:20.000 people^17^ worldwide. It poses additional technical difficulties during abdominal operations and in port placement during laparoscopy due to the mirror image of normal anatomy^7^. Although laparoscopic cholecystectomy has been pointed out as safe in SIT^1^ ^,^ ^11^ ^,^ ^22^ some additional precautions should be considered during dissection due to the altered anatomy^7^. Multiple major surgical procedures have also been reported in patients with SIT, such as pancreatoduodenectomies^12^ ^,^ ^14^ ^,^ ^18^, common bile duct exploration^15^, resection of choledochal cysts^10^, and liver transplantation^25^, but to the best of our knowledge this is the first case of bile duct injury repair in SIT reported in the literature.

## CASE REPORT

A 42-year-old female patient with no significant previous medical history presented to the emergency room of a local hospital with left upper quadrant abdominal pain and fever started ten days before. On physical examination she had positive Murphy’s sign in the left upper quadrant. Laboratory studies showed leukocytosis and elevated C-reactive protein. Upper abdominal ultrasound revealed situs inversus and findings consistent with cholecystitis in a left-sided placed gallbladder. Chest X-ray demonstrated dextrocardia. A diagnosis of situs inversus totalis and acute cholecystitis was made. According to medical records, the patient was then taken to the operating room for a laparoscopic cholecystectomy. A Parkland V acute cholecystitis was observed, and a bile leak was perceived during the dissection of the hepatocystic triangle. Conversion to open surgery was performed due to suspicion of BDI, which was confirmed intraoperatively. A Roux-en-Y hepaticojejunostomy was performed by the local surgeon, with no report on the level of the lesion or any associated vascular injuries.

**Figure 1 -:**
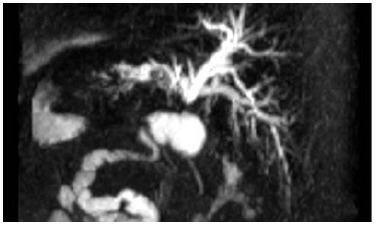
Biliary reconstruction on magnetic resonance cholangiopancreatography demonstrating stenosis of a hepaticojejunostomy.

**Figure 2 -:**
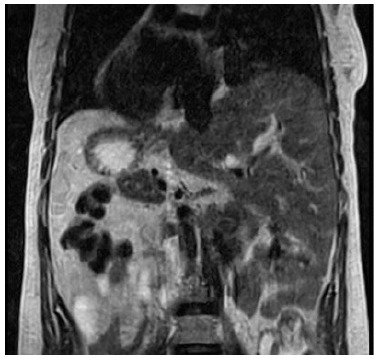
Magnetic Resonance demonstrating situs inversus totalis (dextrocardia, stomach in the right, liver in the left).

After surgery, the patient remained asymptomatic for ten years, and then presented with jaundice, pruritus, acholic stools, and choluria. Due to cholestasis, she was referred to a tertiary center for management. Upon workup she presented with total bilirubin of 1.2 mg/dL, aspartate aminotransferase (AST) of 166, alanine aminotransferase (ALT) of 157, alkaline phosphatase of 727, and gamma-glutamyl transferase (GGT) of 214. Abdominal ultrasound showed SIT, intrahepatic biliary dilation, and absence of hepatic nodules or signs of cirrhosis. Magnetic resonance confirmed intrahepatic biliary dilation and showed stenosis of the previous hepaticojejunostomy (Figures 1 and 2).

**Figure 3 -:**
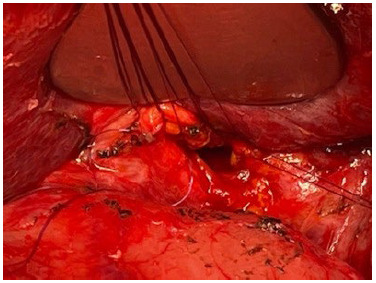
Intraoperative view of Hepp-Couinaud technique. Wide anastomosis performed in the hilar plate side-to-side with interrupted 5-0 polydioxanone.

Due to the young age of the patient, clinical cholestasis, and elevation of canalicular enzymes, a surgical approach was indicated in a multidisciplinary board, with the aim of repairing the stenosis. During surgical exploration a SIT was confirmed intraoperatively, with the liver in the upper left quadrant, the duodenum on the left side, and findings of a previous Roux-en-Y hepaticojejunostomy. The previous stenotic hepaticojejunostomy was undone and remade with the Hepp-Couinaud technique ^28^ high in the hilar plate with a wide opening in the hepatic confluence towards the left hepatic duct (Figure 3). An abdominal drain was left close to the anastomosis. The previous Roux limb was maintained. Postoperative recovery was uneventful, the drain was removed on the seventh post-operative day, and the patient was asymptomatic during follow up, with normal bilirubin and canalicular enzymes, and no further episodes of cholestasis nor cholangitis. The patient signed informed consent for the authors to prepare this report.

## DISCUSSION

To the best of our knowledge, this is the first report of a bile duct injury and repair in a patient with SIT in the literature.

Despite it has been reported that SIT is not a contraindication to laparoscopic cholecystectomy^1^ ^,^ ^11^ ^,^ ^13^, it certainly poses an additional difficulty to both diagnosis and treatment of many conditions, including acute cholecystitis, due to the mirroring of all the abdominal structures^7^_._ Thus, SIT possibly contributed to the BDI presented in this case.

It is worth mentioning that, in case of an intraoperatively diagnosed BDI, in the absence of expertise in hepato-pancreato-biliary (HPB) surgery, it is recommended by the World Society of Emergency Surgery guidelines the placement of a drain and referral to a specialized center for proper management of the lesion^5^. This can avoid performing the hepaticojejunostomy in an ischemic portion of the common bile duct, or in a portion with thermal injury or fibrotic tissue, and therefore, prevent the failure of the anastomosis. It has already been demonstrated that on-table repair by non-HPB specialists appears to be an independent risk factor for biliary strictures, recurrent cholangitis, revision surgery, and overall morbidity^9^ ^,^ ^19^. On the other hand, early referral can decrease the incidence of strictures and postoperative complications^27^, as well as the risk of litigation^2^. The patency of the anastomosis in the long term is larger when performed by an HPB surgeon^16^. Regarding the timing of the repair, it should be considered until eight days after the lesion or after six weeks. For patients presenting between eight days and six weeks, adequate sepsis control and delayed repair should be preferred^6^.

Considering that BDI usually affects patients at a young age, the long-term results should be kept in mind, as the life expectancy of these patients are long. In this matter, for achieving a better repair, the anastomosis should be performed with the Hepp-Couinaud technique at the hilar plate level to avoid the region with thermal injury, to get a better vascularization of the anastomosis, and to allow a wide side-to-side anastomosis in the left bile duct down to the bifurcation^13^ ^,^ ^28^. This is a technically demanding procedure, but when performed according to the Hepp-Couinaud technique by specialized HPB surgeons, it could achieve long-term patency higher than 85%^13^.

In this case, the SIT added some degree of difficulty to the procedure, as the whole anatomy of the patient was mirrored. Extra care had to be taken when dissecting the hepatic hilum or opening the biliary ducts in order not to mistake the sides of the structures.

## CONCLUSIONS

BDI is a severe complication of cholecystectomy. Anatomical variants can increase the risk of BDI. When intraoperatively diagnosed by a non-surgeon specialist, the best option is to place a drain in the right-upper quadrant and refer the patient to a specialized center, where it can be properly managed. SIT certainly increases the difficulty of both diagnosis and surgical procedures.
